# Can *Zymomonas mobilis* Substitute *Saccharomyces cerevisiae* in Cereal Dough Leavening?

**DOI:** 10.3390/foods7040061

**Published:** 2018-04-16

**Authors:** Alida Musatti, Chiara Mapelli, Manuela Rollini, Roberto Foschino, Claudia Picozzi

**Affiliations:** Dipartimento di Scienze per gli Alimenti, la Nutrizione, l’Ambiente, Università degli Studi di Milano, 20133 Milan, Italy; alida.musatti@unimi.it (A.M.); chiara.mapelli1@unimi.it (C.M.); manuela.rollini@unimi.it (M.R.); roberto.foschino@unimi.it (R.F.)

**Keywords:** *Zymomonas mobilis*, *Lactobacillus sanfranciscensis*, sourdough, dough leavening, bakery products, *Saccharomyces cerevisiae*, anti-*S. cerevisiae* antibodies

## Abstract

Baker’s yeast intolerance is rising among Western populations, where *Saccharomyces cerevisiae* is spread in fermented food and food components. *Zymomonas mobilis* is a bacterium commonly used in tropical areas to produce alcoholic beverages, and it has only rarely been considered for dough leavening probably because it only ferments glucose, fructose and sucrose, which are scarcely present in flour. However, through alcoholic fermentation, similarly to *S. cerevisiae*, it provides an equimolar mixture of ethanol and CO_2_ that can rise a dough. Here, we propose *Z. mobilis* as a new leavening agent, as an alternative to *S. cerevisiae*, overcoming its technological limit with different strategies: (1) adding glucose to the dough formulation; and (2) exploiting the maltose hydrolytic activity of *Lactobacillus sanfranciscensis* associated with *Z. mobilis*. CO_2_ production, dough volume increase, pH value, microbial counts, sugars consumption and ethanol production were monitored. Results suggest that glucose addition to the dough lets *Z. mobilis* efficiently leaven a dough, while glucose released by *L. sanfranciscensis* is not so well fermented by *Z. mobilis*, probably due to the strong acidification. Nevertheless, the use of *Z. mobilis* as a leavening agent could contribute to increasing the variety of baked goods alternative to those leavened by *S. cerevisiae*.

## 1. Introduction

In the last decades, the research in human nutrition has aimed both at improving food safety and demonstrating new healthy properties of foods or ingredients. Particularly in the grain cereals area, great attention has been paid to the study of sourdough microbial ecology [1,2] and to the positive effects of lactic acid bacteria (LAB) and yeast fermentation on the technological characteristics of dough. Therefore, several contributions have led to the enhancement of baked products by using a sourdough technology, which also matches consumer’s choices in terms of their preference towards the valorisation of traditional products that can be certified [3]. Organic acid production impacts on sourdough texture and product shelf-life, with acetic acid also displaying anti-ropiness and antifungal activities [4,5]. Acidification also helps to activate endogenous cereal proteases that release peptides and amino acids related to flavour formation [6]. The production of bacteriocins allows microorganisms to control the sourdough ecosystem [7], while the synthesis of homo-polysaccharides delays firmness and staling [6]. Sourdough fermentation can also have positive nutritional implications by biodegrading phytates, thus increasing mineral bioavailability, and by lowering the glycaemic response to the consumption of baked goods [6].

Nevertheless, adverse food reactions, such as baker’s yeast intolerance, have recently been increasing among Western population [8]. Apart from in well-known alcoholic beverages, such as beer, wine and cider, and baked goods, *S. cerevisiae* is also used in savoury spreads, as a food supplement in ‘multi-vitamin’ preparations and ‘probiotics’ in animal feed [9], and even in vaccine production [10]. It is therefore clear that we are often exposed to yeast parietal components [11].

Several studies report that an adverse response to baker’s yeast occurs in a proportion of patients with Inflammatory Bowel Disease (IBD). In particular, in patients with Crohn’s disease (CD), *S. cerevisiae* is recognized as an antigen, and anti-*S. cerevisiae* antibodies (ASCAs), directed against the cell wall mannan (phosphopeptidomannan) of yeast, have been identified as an important serological marker of this pathogenesis. However, the determination of ASCAs is also reliable in other autoimmune disorders besides CD [12]. Environmental factors such as food antigens may play an important role in the pathogenesis of autoimmune disorders [10,13] and obesity [9]. Although there is scarce literature on allergy-hypersensitivity to yeasts, some clinical conditions might benefit from reduced exposure to these microorganisms [14].

Based on these considerations, the study of new microbial resources to be applied in leavened goods may be considered of actual relevance; in this context, the possibility of replacing *S. cerevisiae* is noteworthy. The use of *Zymonomas mobilis* as leavening agent can contribute to an increase in the variety of bakery products alternative to those leavened by yeast in order to meet the specific demands of consumers. *Z. mobilis* can therefore be an interesting candidate to create a new food area of yeast-free baked goods. This bacterium is commonly used in tropical areas as a fermenting agent of plant saps to obtain alcoholic beverages such as pulque [15]. *Z. mobilis* ferments only glucose, fructose and sucrose, and through alcoholic fermentation it provides an equimolar mixture of ethanol and CO_2_ that can theoretically leaven a dough [16], just like *S. cerevisiae*. The narrow range of fermentable substrates is a technological limit of *Z. mobilis* vs *S. cerevisiae* that may be overcome by: (1) adding a fermentable sugar to the dough formulation; or (2) exploiting maltose hydrolytic activity of *Lactobacillus sanfranciscensis* associated with *Z. mobilis.* This unconventional association has been investigated as a model system (higher cell concentration and leavening temperature, shorter leavening time) in a previous paper [17]. The present research aims to compare *Z. mobilis* leavening performance when glucose is added to the dough both with its fermentative ability when *Z. mobilis* is in association with *L. sanfranciscensis* and in doughs formulated and processed similarly to a type I sourdough.

## 2. Materials and Methods 

### 2.1. Microorganisms and Maintenance

*Z. mobilis* subs. *mobilis* type strain DSM 424 (DSMZ: Deutsche Sammlung von Mikroorganismen und Zellkulturen GmbH) and *L. sanfranciscensis* DSM 20663 were used in this study.

*Z. mobilis* was maintained in liquid DSM medium, while biomass production was carried out in liquid IC G20 medium (as previously reported) [16]. Both media contain bacto-peptone (Costantino SpA, Turin, Italy) 10 g/L and glucose (Sigma Aldrich, St. Louis, MO, USA) 20 g/L, while they differ for yeast extract (Costantino SpA) 10 g/L present in DSM medium and of casein enzymatic hydrolysate (Costantino SpA) 10 g/L in IC G20. For both media, the pH was set at 6.8, and sterilization occurred at 112 °C for 30 min.

*L. sanfranciscensis* was maintained and cultivated in MRSm medium as reported elsewhere [17]. Cultures were incubated at 30 °C in stationary conditions for 16–24 h. Stock cultures of both microorganisms were stored at −80 °C in the same media (DSM for *Z. mobilis* and MRSm for *L. sanfranciscensis*) added with 20% (*v*/*v*) glycerol (VWR International, Leuven, Belgium).

### 2.2. Biomass Production 

*Z. mobilis* was cultured in 1 L flasks containing 600 mL of liquid IC G20 medium, inoculated with 5% (*v*/*v*) of a 9 h pre-culture grown in DSM medium. *L. sanfranciscensis* was grown in 1 L flasks containing 600 mL of MRSm medium, inoculated with 2% (*v*/*v*) of a 24 h pre-grown culture in the same medium. Cultures were incubated at 30 °C in stationary conditions for 16 h for *Z. mobilis* and 24 h for *L. sanfranciscensis*.

The determination of the cell biomass was performed by spectrophotometric measurement (OD 600 nm, 6705 UV-Vis Spectrophotometer, Jenway, UK). For each strain, at 16 h in the case of *Z. mobilis* and 24 h for *L. sanfranciscensis*, a calibration curve was built (OD 600 vs. CFU (colony-forming unit)/mL) to determine the proper culture volume to add in the dough preparation (cell concentration expressed as Log CFU/g dough).

### 2.3. Dough Production and Analytical Determinations

Doughs were prepared with 333 g of a commercial type 0 Manitoba wheat flour (Simec SpA, Santa Giusta, Oristano, Italy) and 167 mL of distilled water, with or without addition of 1 or 5% (*w*/*w* flour) glucose. *Z. mobilis* was added alone (7 Log CFU/g dough) or with *L. sanfranciscensis* (5 Log CFU/g dough) yielding to 100:1 ratio *Zymomonas*:*Lactobacillus* cells. Ingredients were mixed in a food mixer (CNUM5ST, Bosch, Stuttgart, Germany) at speed 1 for 6 min. The dough was divided into 3 sections, treated as follows and then incubated at 26 °C:

- 400 g, inserted into a 1 L graduate cylinder to evaluate the dough volume increase up to 24 h of leavening;
- 25 g, inserted into a double chamber flask connected with a graduate burette filled with acidified water, to evaluate the total amount of CO_2_ produced during leavening [16];
- The remaining sample was left to leaven into a Becker; samples were taken at appropriate intervals to determine dough pH, microbial counts and to carry out HPLC (high performance liquid chromatography) analysis.

Each analysis was performed at 0, 8, 16 and 24 h of leavening time.

### 2.4. Evaluation of Dough Volume Increase and Total CO_2_ Production

The increase in the dough volume (mL) was evaluated at appropriate time intervals through the record of the level reached by the dough inside the graduate cylinder. CO_2_ production (mL) was monitored by measuring the level reached by the liquid present inside the burette connected to the double chamber flask.

### 2.5. Determination of the Microbial Populations in Doughs

Approximately 10 g of dough sample were decimally diluted in sterile peptone water (10 g/L Bacto-peptone (Costantino SpA), pH 6.8) and homogenized in a Stomacher 400 Circulator (Seward, Worthing, UK) for 5 min at 260 rpm. The appropriate dilutions were plated onto MRSm agar (MRSm broth added with agar 15 g/L) for the determination of *L. sanfranciscensis* population, as well as onto DSM agar (DSM broth added with agar 15 g/L) for *Z. mobilis*. Plates were incubated at 30 °C for 3 d in anaerobic conditions. Aerobic bacterial count (ABC) was determined by pour plating in Tryptic Soy Agar (TSA, Scharlab, Barcelona, Spain) after incubation at 30 °C for 48–72 h. The enumeration of yeasts and moulds were carried out in Yeast Glucose Chloramphenicol Agar (YGC-Scharlab, Barcelona, Spain) plates after incubating at 25 °C for 3–5 day.

### 2.6. HPLC Analyses and pH Monitoring

Maltose and glucose consumption, as well as ethanol production during leavening, were measured through an HPLC system (L 7000, Merck Hitachi, Tokyo, Japan) as reported by Musatti et al. [17]. Briefly, 4 mL of homogenized dough samples were centrifuged (Eppendorf 5804 (Hamburg, Germany), 10,600× *g*, 10 min) and supernatants were filtered (0.45 µm syringe filter, VWR International, Radnor, PA, USA) before HPLC analysis. Data refer to 1 g dough (mg/g dough).

Dough pH was monitored at different intervals on the integral undiluted dough sample (pH-meter Eutech Instruments pH 510, Toronto, ON, Canada).

### 2.7. Statistical Analysis

All samples were prepared and analysed at least in triplicate. The effect of two factors, such as % glucose addition or *L. sanfranciscensis* co-inoculation, on some fermentation parameters were investigated by ANOVA according to the general linear model. Results of microbiological counts were transformed in the respective decimal logarithms to match a normal distribution of values. Data were processed with Statgraphic R Plus 5.1 for Windows (StatPoint, Inc., Herndon, VA, USA). When the effect was significant (*p* < 0.05), differences between means were separated by LSD test of multiple comparisons.

## 3. Results and Discussion

### 3.1. Trials with Glucose Addition into Dough

Dough samples were prepared with or without glucose addition (1% and 5% *w*/*w*). When glucose was not added, *Z. mobilis* fermented only the glucose amount naturally present in the flour (around 2.01 ± 0.59 mg/g dough). However, even if there are some hydrolytic enzymatic activities in the dough due to the presence of endogenous amylases, the low glucose concentration does not allow adequate CO_2_ production to obtain a suitable dough volume increase by *Zymomonas*, especially in the first times of incubation.

**Figure 1:**
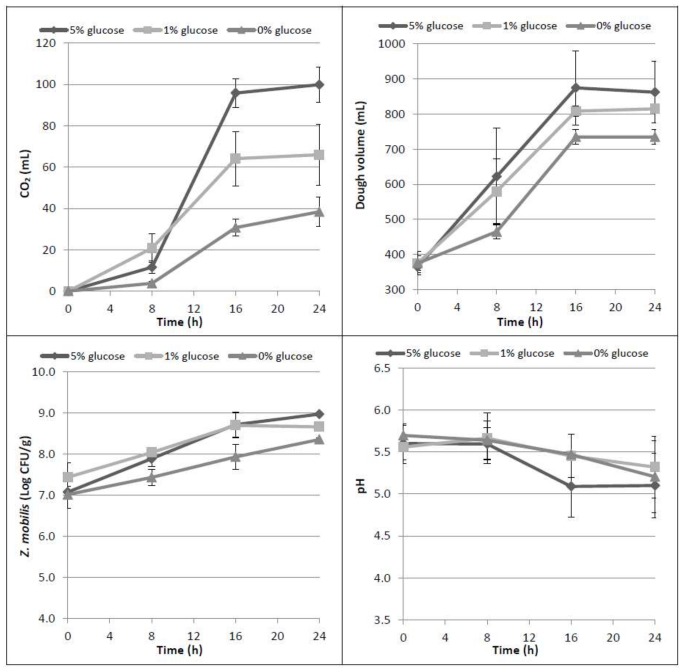
Time course of CO_2_ production (mL), dough volume increase (mL), microbial growth of *Z. mobilis* (Log CFU/g) and dough pH in the three tested conditions (0%, 1%, 5% *w*/*w* glucose).

The need to add a fermentable carbon source to the flour, in order to obtain a leavening of the dough, had already been highlighted in a previous work [16]. Actually, the results confirmed that the addition of glucose increases the CO_2_ production (*p* = 0.001), and that in the three tested conditions mean values became statistically different at 16 h leavening time (*p* = 0.007) (Figure 1). Similarly, the addition of glucose allowed the doubling of the dough volume within the considered incubation time. As expected, the highest CO_2_ production is related to the highest dough volume increase; in particular, with 5% glucose, the mean value of dough volume reached more than 850 mL (*p* = 0.019) with respect to an average of 815 or 735 mL with 1% or without glucose addition, respectively. CO_2_ production was also related to bacterial growth; when no glucose is added, *Z. mobilis* grew approximately 1.3 Log CFU/g in 24 h, and around 1.6–2 Log CFU/g in the presence of 1% and 5% glucose, respectively. The performances obtained in dough samples with the two glucose concentrations were not significantly different between them, but both were statistically different from those obtained without glucose (*p <* 0.001).

Results from HPLC analysis confirmed the increase of maltose during leavening time (*p* < 0.001) due to hydrolytic activity of the flour amylases and *Z. mobilis* inability to use this sugar (Table 1). At up to 8 h of incubation, the ethanol formation was not statistically different (*p* = 0.414), even if the three tested conditions had different levels of glucose. Then, glucose was mainly consumed between 8 and 16 h, producing CO_2_ and ethanol in higher amounts in samples to which 5% glucose was added, as expected.

### 3.2. Bacterial Association Z. mobilis-L. sanfranciscensis

The association of *Zymomonas* with lactic acid bacteria has already been described in various food products, especially in some fermented drinks [18,19,20,21]. From this perspective, the possibility of obtaining a gradual glucose release in the dough exploiting the maltose hydrolytic activity of *Lactobacillus sanfranciscensis* was investigated [17].

**Figure 2:**
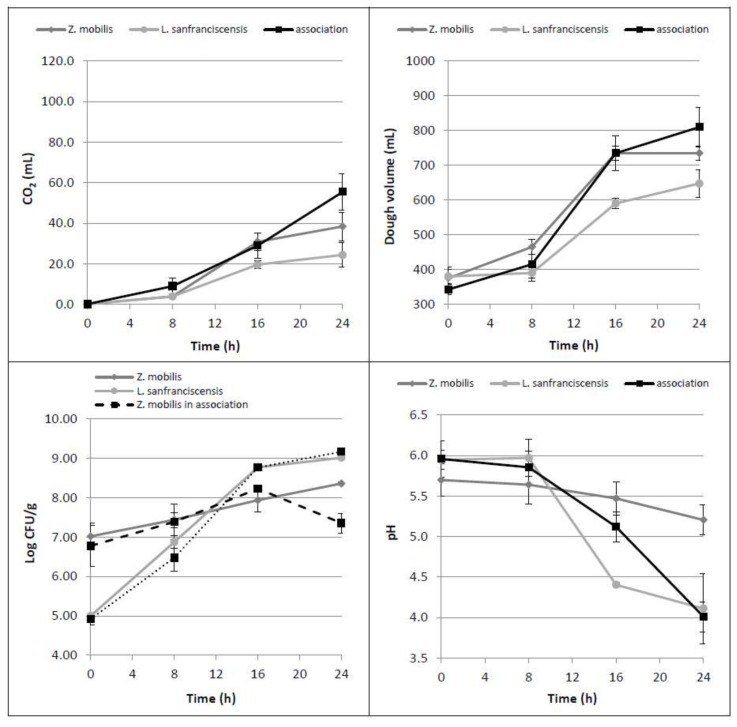
Time course of CO_2_ production (mL), dough volume increase (mL), microbial growth (Log CFU/g) as well as dough pH, in doughs leavened by *Z. mobilis* and *L. sanfranciscensis* alone or by their association.

When *L. sanfranciscensis* and *Z. mobilis* were inoculated together, the mean values of CO_2_ production and dough volume increase did not significantly differ from those obtained with the use of *Z. mobilis* alone (Figure 2). Dealing with the single leavened samples, dough volumes at 16 and 24 h were found to be statistically different from those observed by using *L. sanfranciscensis* alone (*p* = 0.023 and 0.024, respectively). These results indicate that the contribution of the two microorganisms in association is not additive. Respect to the trials performed with glucose addition, in which the dough volumes nearly doubled in the first 8 h, the dough volume increased less than 20% in the case of the microbial association. As regards the trends of acidification and bacterial counts during the incubation time, the obtained data proved to be strongly affected by the presence of *L. sanfranciscensis*: the pH decreased to values of around 4 and the LAB growth (plus 4 Log CFU/g) was not influenced by the presence of *Zymomonas*. On the contrary, *Z. mobilis*, when grown in association with *L. sanfranciscensis*, statistically reduced (*p* = 0.002) its cell concentration from 16 h leavening onward. This behavior is probably due to the strong acidification of the medium produced by *L. sanfranciscensis*, able to affect both *Z. mobilis* vitality and fermentation ability.

HPLC data confirmed that *L. sanfranciscensis* consumed maltose (*p* = 0.012) and released glucose (*p* = 0.003) in the dough [4], that it is not totally consumed by *Z. mobilis* (Table 2).

In summary, these results highlight that when inoculated alone, *Z. mobilis* is able to consume all the glucose present in a dough, while when coupled with *L. sanfranciscensis*, its fermentative performance decreases. Furthermore, the presence of *L. sanfranciscensis* did not lead to a significant ethanol yield increase, even if it can consume the available maltose in flour and release glucose.

## 4. Conclusions

The results obtained demonstrate that *Z. mobilis* is able to efficiently leaven a dough when glucose is present in the dough formulation. On the other hand, although the metabolic activities of LAB have positive effects on the structural and sensorial properties of the baked product [5,6], the traditional back-slopping sourdough technology [22] cannot be proposed due to the accelerated acidification of the dough impairing the growth of *Zymomonas.* In fact, preliminary trials have evidenced that, independently of the initial cell ratio between the two bacteria, *L. sanfranciscensis* always became the prevailing microbial population. This disproportion with *Z. mobilis* increased with refreshments, thus giving strongly acidified and poorly leavened doughs.

Future trials will be aimed at investigating microbial association with other LAB or the use of dough formulations naturally enriched of sugars fermentable by *Z. mobilis*. In this context, sucrose can also be considered an interesting alternative to glucose; nevertheless, the strain leavening performance with this carbon source has to be evaluated.

## Figures and Tables

**Table 1:** Maltose, glucose and ethanol concentrations (expressed in terms of mg/g dough, mean and standard deviation (St. dev.)) present at 0, 8, 16 and 24 h in doughs leavened by *Z. mobilis* with 0%, 1%, 5% (*w*/*w*) of glucose added respect to the flour.

Glucose (% *w*/*w* flour)	Time (h)	Maltose (mg/g)		Glucose (mg/g)		Ethanol (mg/g)	
		Mean	St. dev.	Mean	St. dev.	Mean	St. dev.
0	0	10.50	0.96	1.96	0.06	0.00	0.00
	8	14.59	1.67	1.12	0.13	1.14	0.13
	16	17.71	3.16	0.23	0.33	2.79	0.49
	24	15.69	2.65	0.00	0.00	3.38	0.33
1	0	10.55	1.11	8.72	0.41	0.00	0.00
	8	15.48	5.22	3.42	0.62	1.84	0.41
	16	19.00	1.59	1.23	0.54	4.03	1.02
	24	21.43	4.49	0.92	0.44	3.96	0.77
5	0	8.33	0.95	35.72	2.95	0.00	0.00
	8	15.72	3.29	34.56	1.62	0.72	1.01
	16	18.57	2.84	2.66	1.48	9.73	0.13
	24	20.80	2.92	1.46	0.40	13.65	2.46

**Table 2:** Maltose, glucose and ethanol concentrations (expressed in terms of mg/g dough, mean and standard deviation) present at 0, 8, 16 and 24 h in doughs leavened by *Z. mobilis*, *L. sanfranciscensis* and their association.

Microorganism	Time(h)	Maltose (mg/g)		Glucose (mg/g)		Ethanol (mg/g)	
		Mean	St. dev.	Mean	St. dev.	Mean	St. dev.
*Lactobacillus sanfranciscensis* (5 Log CFU/g)	0	11.33	1.46	2.08	1.01	0.00	0.00
	8	16.68	0.66	3.20	0.82	0.00	0.00
	16	9.43	0.60	4.01	0.28	0.00	0.00
	24	10.80	0.80	4.54	0.52	0.00	0.00
*Zymomonas mobilis* (7 Log CFU/g)	0	10.50	0.96	1.96	0.06	0.00	0.00
	8	14.59	1.67	1.12	0.13	1.14	0.13
	16	17.71	3.16	0.23	0.33	2.79	0.49
	24	15.69	2.65	0.00	0.00	3.38	0.33
*L. sanfranciscensis* coupled with *Z. mobilis* (5–7 Log CFU/g)	0	8.96	1.82	1.04	0.56	0.00	0.00
	8	14.21	2.70	0.92	0.31	0.75	0.28
	16	13.58	0.94	0.56	0.79	3.73	0.70
	24	12.33	2.76	0.82	0.35	4.91	1.23
